# A novel nonsense mutation in the tyrosinase gene is related to the albinism in a capuchin monkey (*Sapajus apella*)

**DOI:** 10.1186/s12863-017-0504-8

**Published:** 2017-05-05

**Authors:** Felipe Tadeu Galante Rocha de Vasconcelos, Einat Hauzman, Leonardo Dutra Henriques, Paulo Roney Kilpp Goulart, Olavo de Faria Galvão, Ronaldo Yuiti Sano, Givago da Silva Souza, Jessica Lynch Alfaro, Luis Carlos de Lima Silveira, Dora Fix Ventura, Daniela Maria Oliveira Bonci

**Affiliations:** 1 Departamento de Psicologia Experimental, Instituto de Psicologia, Universidade de São Paulo, Av. Professor Mello Moraes 1721 Bloco A Sala D9 – Butantã, São Paulo, SP Brazil 05508-030; 2 Instituto Israelita de Ensino e Pesquisa Albert Einstein, Hospital Israelita Albert Einstein, São Paulo, São Paulo Brazil; 3 Núcleo de Teoria e Pesquisa do Comportamento, Universidade Federal do Pará, Rua Augusto Corrêa, 01 - Guamá, Belém, PA Brazil 66075-110; 4 Departamento de Oftalmologia, Santa Casa de Misericórdia de São Paulo, São Paulo, São Paulo Brazil; 5 Instituto de Ciências Biológicas, Universidade Federal do Pará, Rua Augusto Corrêa, 01 - Guamá, Belém, PA Brazil 66075-110; 6 Núcleo de Medicina Tropical, Universidade Federal do Pará, Belém, Pará Brazil; 7 Institute for Society and Genetics, University of California Los Angeles, Box 957221, 3360 LSB, Los Angeles, CA USA 90095-7221; 8 Department of Anthropology, University of California Los Angeles, Los Angeles, CA USA

**Keywords:** Albino, OCA, TYR gene, *Sapajus apella*, Stop codon

## Abstract

**Background:**

Oculocutaneous Albinism (OCA) is an autosomal recessive inherited condition that affects the pigmentation of eyes, hair and skin. The OCA phenotype may be caused by mutations in the tyrosinase gene (TYR), which expresses the tyrosinase enzyme and has an important role in the synthesis of melanin pigment. The aim of this study was to identify the genetic mutation responsible for the albinism in a captive capuchin monkey, and to describe the TYR gene of normal phenotype individuals. In addition, we identified the subject’s species.

**Results:**

A homozygous nonsense mutation was identified in exon 1 of the TYR gene, with the substitution of a cytosine for a thymine nucleotide (C64T) at codon 22, leading to a premature stop codon (R22X) in the albino robust capuchin monkey. The albino and five non-albino robust capuchin monkeys were identified as *Sapajus apella*, based on phylogenetic analyses, pelage pattern and geographic provenance. One individual was identified as *S. macrocephalus*.

**Conclusion:**

We conclude that the point mutation C64T in the TYR gene is responsible for the OCA1 albino phenotype in the capuchin monkey, classified as *Sapajus apella*.

**Electronic supplementary material:**

The online version of this article (doi:10.1186/s12863-017-0504-8) contains supplementary material, which is available to authorized users.

## Background

Albinism is a heterogeneous disorder, characterized by the absence or loss of melanin pigmentation in either skin, hair or eyes. This condition is considered rare in wild populations [1] possibly due to the reduction in camouflage [2, 3] and to its role in a number of pathologies that can compromise an animal’s fitness. Several pathological conditions are associated with albinism, such as immunological [4–6], auditory [7, 8] and visual deficiencies, e.g., nystagmus, absence of fovea, alteration of the optic chiasm decussation, reduction of rod density, photophobia, strabismus, iris transilumination and reduction of visual acuity [5, 9–13].

The biochemical pathway of melanin production is catalyzed by the tyrosinase enzyme, which is expressed by the tyrosinase gene (TYR) [14]. A deficiency in tyrosinase production leads to the total or partial absence of melanin, resulting in the Oculocutaneous Albinism Type 1 (OCA1) [15]. Cases of OCA1 caused by mutations in the TYR gene have been reported in several mammalian species, e.g., in rabbits [16], cattle [17], rats [18], cats [19], ferrets [20], whales [21] and buffalos [22]. Among primates a genetic mutation in the TYR gene was described in two unrelated individuals of rhesus monkey (*Macaca mulatta*), with phenotypes similar to human OCA1 [23]. Also in primates, a mutation in the SLC45A2 gene was indicated to cause the Type 4 Oculocutaneous Albinism (OCA4) phenotype in one specimen of gorilla (*Gorilla gorilla*) [24].

**Fig. 1:**
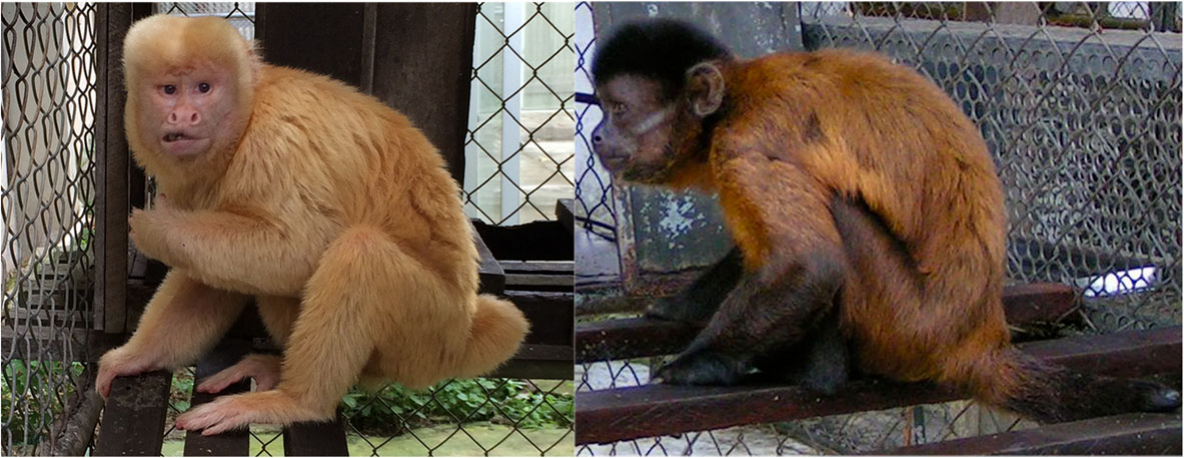
The albino capuchin monkey *Sivuca* (*left*) and a normal phenotype capuchin monkey *Smeagol* (*right*), with the expected pelage pattern of *Sapajus apella*, at the Experimental School for Primates (EEP), Universidade Federal do Pará (UFPA), Belém, Brazil

In this study, we investigated the genetic causes of albinism in a capuchin monkey, named *Sivuca*, held in the Experimental School for Primates (EEP), at the Universidade Federal do Pará (UFPA), Belém, Brazil. This adult male (Fig. 1) was found abandoned in the streets of Breves, an Amazon riverside town, handed in to the Brazilian Institute of Environment and Natural Resources (IBAMA), and transferred to the EEP, where it received medical treatment and is currently housed. Young wild animals are prized as exotic pets and are subject to animal trafficking. When these animals grow up into adults they can become dangerous and are often abandoned. *Sivuca* had his canine teeth filed down by the owners and his tail cut off, probably in a trap. Due to his albinism and history we cannot ascertain where he came from and he cannot be reintroduced into nature. However, *Sivuca* has adapted well to the captive housing conditions and provides us with an extremely valuable opportunity to identify albinism gene alteration in a species closely related to humans. Studies on different aspects of the albinism in other species, especially in primates, give us important insights into the mechanisms and development of albinism in humans. The description of the tyrosinase gene mutation may contribute to elucidate the underlying machinery of cell pigmentation and its influence on visual system development and the functioning of other systems affected by albinism. Thus, *Sivuca* represents a good model for a better understanding of this complex disorder.

Capuchin monkeys are divided in two genera, the gracile (*Cebus*) and the robust capuchin (*Sapajus*) [25]. Species identification for capuchins demands careful morphological evaluation, information about provenance and molecular phylogenetic analysis [26]. The absence of regular fur coloration and lack of information on capture location makes it impossible to identify the species to which *Sivuca* belongs without molecular phylogenetic analysis. Thus, the objectives of this study were to identify the mutation responsible for the albino phenotype in the capuchin monkey *Sivuca*, and to describe the tyrosinase gene sequence of normal phenotype subjects from the same species. In addition we used molecular phylogenetic analysis for dentification of the subject’s species,

## Methods

### Samples

We analyzed the TYR coding region from genomic DNA using blood samples collected from the albino capuchin monkey (Fig. 1) and from six normal phenotype adult capuchin monkeys (five males and one female), held in the EEP and provisionally identified as *Sapajus apella* (see below). The animal procedures were in accordance with ethical principles of animal management and experimentation established by the Brazilian Animal Experiment College (COBAE), and the study was approved by the Ethical Committee of the UFPA (CEPAE/UFPA/#040–2015).

### DNA extraction and PCR amplification for genetic sequencing

DNA extraction was performed using the Gentra Puregene Blood kit (Gentra Systems, Inc., Minneapolis, Minn., USA), according to the protocol supplied by the manufacturer. Each of the five exons of the TYR gene was amplified using the primers described in Table 1. Primers for exons 1 and 2 were obtained from Preising et al. [27], for amplifying human TYR gene. Primers for exons 3, 4 and 5 were designed on Primer 3 (v. 0.4.0) [28], based on the TYR sequence of the black-capped squirrel monkey *Saimiri boliviensis* (GenBank accession number XM_003935082.1). Polymerase Chain Reactions (PCRs) were carried out using High Fidelity Platinum Taq Polymerase, 10x High Fidelity Buffer, MgCl_2_ (Invitrogen), 10 mM GeneAmp dNTPs (Applied Biosystems, Inc.) and 20 mM primers in 50 μl reactions. The PCR conditions were an initial denaturation at 94 °C for 1 min; 37 cycles of 15 s at 94 °C, 30 s at the annealing temperature (Table 1) and 30 s at 72 °C; and a final extension at 72 °C for 7 min. PCR products were visualized by electrophoresis on agarose gel (1%) and purified with Illustra GFX^TM^ PCR DNA and Gel Band Purification Kit (GE Healthcare, Little Chalfont, Buckinghamshire, UK). The PCR products were directly sequenced in both directions with BigDye® Terminator v3.1 Cycle Sequencing Kit (Applied Biosystems, Inc.) and the 3500 Applied Biosystems Sequencer. Nucleotide sequences were analyzed and aligned in BioEdit v7.0.9.0 [29], using ClustalW, and the alignment was visually confirmed.

**Table 1:** Primers used to amplify the TYR gene, the D-loop and Cytochrome b

Gene	Primer	Annealing temperature	Nucleotide sequence
^1^TYR Exon 1	*TYR11Fw*	59	5' CCAATTAGCCAGTTCCTGCAGA 3'
	*TYR11Rv*		5' CACAGTTGAATCCCATGAAGTTGC 3'
	*TYR12Fw*	57	5' TATAATAGGACCTGCCAGTGCTCTG 3'
	*Tyr12Rv*		5' AATGTCTCTCAAGATTTCAGATCCC 3'
	*Tyr13Fw*	60	5' TGTGTCAATGGATGCACTGCTT 3'
	*Tyr13Rv*		5' AGAAGTGATTGTTAAGGTTCCTCCC 3'
^1^TYR Exon 2	*Tyr2Fw*	60	5' TTGTTTAACATGAGGGTGTTTTGTACAG 3'
	*Tyr2Rv*		5' GGACTTTGGATAAGAGACTGTAAATATG 3'
TYR Exon 3	*Tyr3SbFw*	59	5' TCCATTTACTGGGATAGCAGATG 3'
	*Tyr3SbRv*		5' GTGAAGAAGGAAGATGGGATCAT 3'
TYR Exon 4	*Tyr4SbFw*	59	5' GAAGGCATCGCCCTCTTCTA 3'
	*Tyr4SbRv*		5' AGGTAGCTATAGTCATAGCCCAGA 3'
TYR Exon 5	*Tyr5SbFw*	60	5' CCCAGACTCTTTTCAAGACTAACATT 3'
	*Tyr5SbRv*		5' AATAAAGATGGGGCCAATAAAAA 3'
^2^D-loop	*L*	60	5' GGCCTTGTAAACCGGAAAAGG 3'
	*R*		5' GAAAGGCTAGGACCAAACCTG 3'
^3^CytB	L14724	58	5' CGAAGCTTGATATGAAAAACCATCGTTG 3'
	CIT-REV		5' GAATATCAGCTTTGG 3'

^1^Preising et al. [27]; ^2^Schneider et al. [40]; ^3^Irwin et al. [41]

### Phylogenetic approach for species classification

The capuchin monkey group includes two distinct genera, *Sapajus* and *Cebus*, [30], and species classification within each genus demands more than a simple morphological evaluation because of the highly polymorphic nature of within-species variation in capuchin monkeys [31, 32]. As the captive animals in the study are of unknown provenance, their species affiliation is uncertain. In order to determine the most likely geographic and species affiliation we amplified and sequenced the Cytochrome b and D-loop (GenBank accession numbers KY971495 and KY971496, respectively) regions of the mitochondrial DNA (as described above), using primers described in Table 1. We created an alignment using sequences from Lima et al. [33] that included samples from all currently recognized *Sapajus* species [32] and geographic subclades within species groups [33]. We aligned the sequences from these 63 samples of known species designation with Cytochrome b and D-loop sequences from the albino capuchin *Sivuca* and six other non-albino captive capuchins captured by IBAMA from nearby localities and housed at EEP, for a total of 70 capuchin samples. We aligned and concatenated the data set in Geneious Pro 5.3.4. In our D-loop alignment, we removed a small number of base pairs (bp) that were ambiguously aligned. Our final alignment consisted of a total of 586 bp of Cytochrome b and 585 bp of D-loop. We ran the dataset on Partition Finder v.1.1.1 [34] with linked branch lengths, all models, BIC for model selection, and greedy search parameters to determine the best-fit model of gene evolution for each partition. We recovered the best partitioning scheme as follows: CYTB 1^st^ and 2^nd^ codon positions as HKY + I, CYTB 3^rd^ position as HKY, and D-loop as HKY + I + G. We subsequently ran a MrBayes 3.2 [35] Markov chain Monte Carlo (MCMC) analysis for 1,000,000 generations, with trees sampled every 1,000 generations. We discarded the first 25% of the data as burnin, and used ‘sumt’ to calculate the Bayesian consensus tree, visualized in Figtree v.1.4.0 (available from: http://tree.bio.ed.ac.uk/software/figtree/). GenBank Accession numbers for all sequences are available in Additional file 1. For final species classification the phylogenetic analysis results were compared with an evaluation of the pelage patterns of non-albino individuals [33].

## Results and discussion

### Species identification

**Fig. 2:**
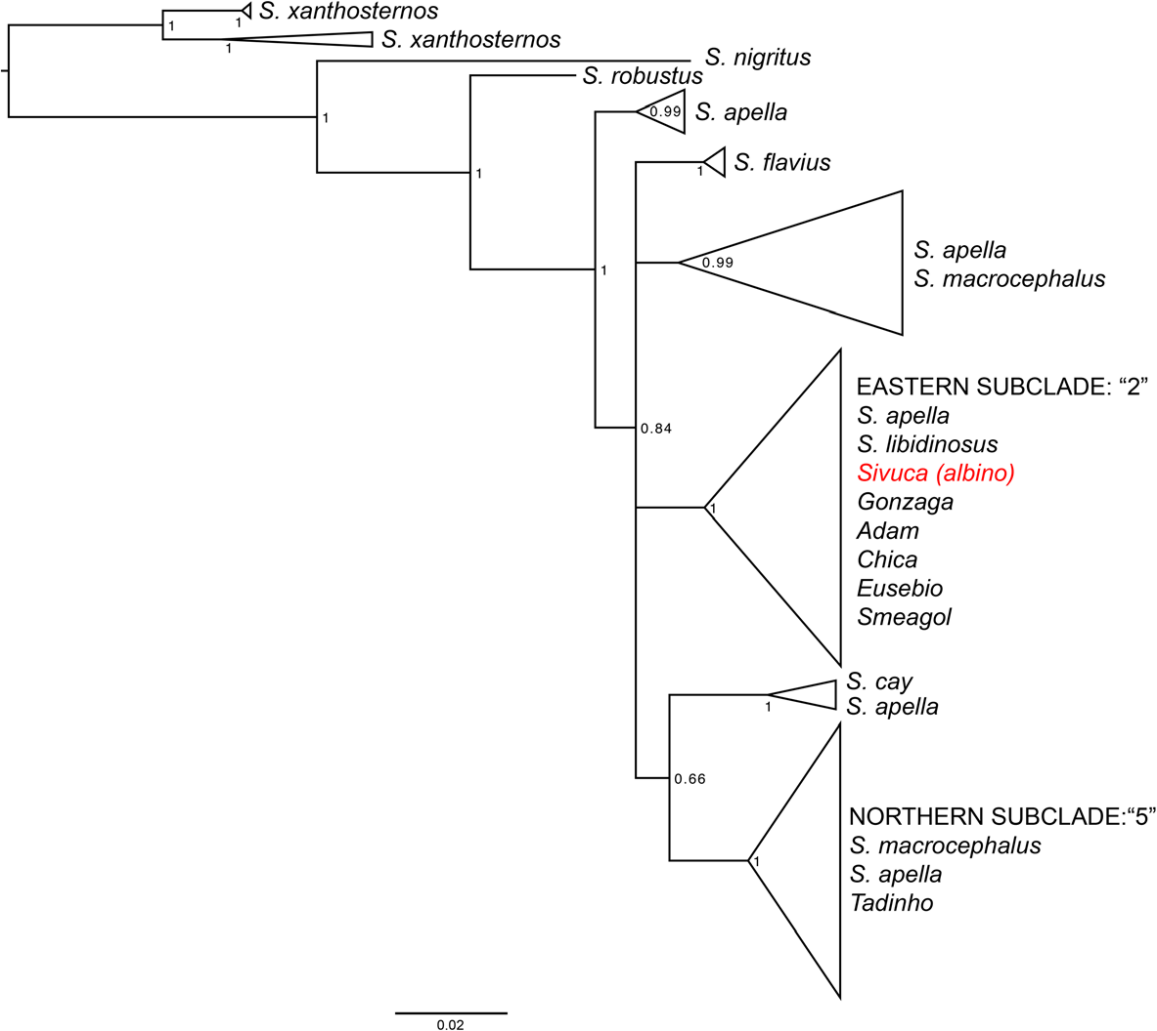
Phylogenetic tree obtained from Bayesian analysis for *Sapajus* species, based on concatenated alignment of Cytochrome b and D-loop region of the mitochondrial DNA, of seven individuals housed at the Experimental School for Primates (EEP), at the Universidade Federal do Pará (UFPA), Belém, Brazil, including the albino *Sivuca* and 63 samples from currently recognized *Sapajus* species [42] and the geographic subclades within species groups [33]. Numbers at nodes represent Bayesian posterior probability. The scale bar represents the number of nucleotide substitutions per site

The albino capuchin monkey *Sivuca* and five non-albino ‘normal’ phenotype captive individuals clustered in a clade with *Sapajus apella* and *Sapajus libidinosus* individuals (Fig. 2), with high posterior probability (pp = 1). This clade corresponds to “*Sapajus* CLADE 4, *Subclade* 2” in the extensive capuchin-wide phylogeny by Lima et al. [33]. This subclade includes both *Sapajus libidinosus* individuals from throughout the Caatinga and Cerrado habitats, as well as Eastern *Sapajus apella* from Tocantins and Tucuruí. Based on the genetic data, the pelage patterns for the non-albino individuals (Fig. 1) and the observation from EEP staff that the monkeys may have originated from the right bank of the Tocantins River, that marks the far eastern end of the distribution for *Sapajus apella*, we classify the genus and species [36, 37] of the albino and normal phenotype capuchin monkeys analyzed in this study as eastern Brazilian *Sapajus apella*. The exception is one individual (*Tadinho*), whose mtDNA sequence formed a clade instead with *S. macrocephalus* and *S. apella* individuals from “*Sapajus* CLADE 4, *Subclade* 5” [33], with high support (pp = 1) (Fig. 2). This is a northern clade of Amazonian *Sapajus* that spans the Amazon River and extends from northern Brazil to the Guianas.

### Capuchin monkey tyrosinase gene

Most studied cases of albinism in human and other mammals are associated with mutations in the TYR gene and therefore, we focused our investigation on this gene in the albino capuchin monkey. This is the first study to describe the tyrosine gene in capuchin monkeys. The TYR coding sequence in mammals consists of five exons. We were able to partially amplify and sequence the five exons of the albino and the non-albino capuchin monkeys, with some gaps between the exon junctions (Additional file 2). No variation was observed among the TYR coding sequences analyzed of the six normal phenotype capuchins. The estimated TYR coding sequence had a total length of 1590 bp that together encode a 530 amino acid TYR protein. The length of the TYR coding sequence varies slightly among species, with 529 amino acids in humans and 530 in the rhesus monkey and the black-capped squirrel monkey (GenBank accession numbers: NM000372.4, XM_001105033.3 and XM_003935082.1, respectively). The TYR coding sequence of normal phenotype *Sapajus apella* capuchin monkey was deposited in GenBank database (KY990734).

### Tyrosinase gene mutation

**Fig. 3:**
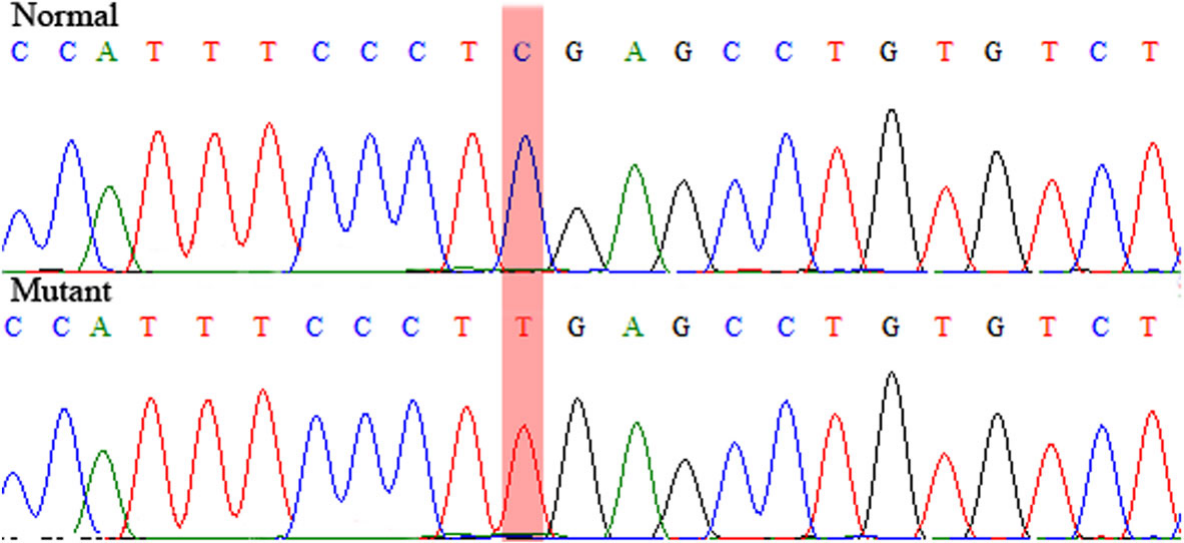
Normal and mutant sequences of the TYR gene in the region of the R22X nonsense mutation of capuchin monkeys *Sapajus apella*

The TYR gene sequences from the albino and normal phenotype capuchin monkeys were aligned and analyzed to determine if there were polymorphisms in the gene sequences. We found no polymorphisms, except a homozygous C-to-T transition at position +64 within the first exon (Fig. 3), which leads to the conversion of an arginine codon (CGA) into a stop codon (TGA) in the albino primate only. This premature stop codon (R22X) produces a truncated TYR protein with only 22 amino acids and has not been described in other species. Notwithstanding, nonsense mutations in exon 1 have been described in human (W128X) [38], rhesus monkey (S184X) [23] and mink (C46X) [39], and all resulted in the OCA type 1 phenotype. Albinism was also associated with frameshift mutations in codon 316 in cattle [17] and at codon 325 in cats, which caused a premature stop codon nine codons downstream of the deletion [19].

## Conclusions

We detected a nonsense mutation (C64T) in exon 1, which generated a premature stop codon (R22X) in the TYR gene of the albino capuchin monkey *Sivuca*. This mutation generates a truncated TYR protein, which is responsible for the OCA1 phenotype in this individual. *Sivuca* was classified as *Sapajus apella*, based on the phylogenetic reconstruction of the D-loop and Cytochrome b regions of mitochondrial DNA. Thus, this study provides the identification and description of a new nonsense mutation causing OCA1 albinism and describes the tyrosinase gene in normal phenotype capuchin monkeys, *Sapajus apella*.

## Additional files

Additional file 1: GenBank accession numbers and geographic coordinates for robust capuchin samples from GenBank used in this study. (PDF 95 kb)

Additional file 2: Alignment of the amino acid sequences of the TYR proteins of human (XM_011542970.2), rhesus monkey (XM001105033.3), marmoset (XM_002754705.2), squirrel monkey (XM_003935082.1) and the capuchin monkey. Identical residues are indicated by dots. The position of the nonsense mutation in the albino capuchin monkey, *Sivuca*, is shown by a star. (TIF 1166 kb)

## Abbreviations

BIC: Bayesian information criterion
EEP: Experimental School for Primates
bp: base pairs
MCMC: Markov Chain Monte Carlo
OCA: Oculocutaneous Albinism
pp: posterior probability
TYR: Tyrosinase

## References

[1] Walter HE (1914). Genetics, an introduction to the study of heredity.

[2] Sazima I, Di-Bernardo M. Albinismo em serpentes neotropicais. Mem Inst Butantan. 1991;53:167–73.

[3] Parsons GJ, Bondrup-Nielsen S (1995). Partial albinism in an island population of Meadow Voles, Microtus pennsylvanicus, from Nova Scotia. Can Field-Naturalist.

[4] Manglani M, Adhvaryu K, Seth B (2004). Griscelli Syndrome - A Case Report.

[5] Summers CG (2009). Albinism: classification, clinical characteristics, and recent findings. Optom Vis Sci.

[6] González Carretero P, Noguera Julian A, Ricart Campos S, Fortuny Guasch C, Martorell SL (2009). Síndrome de Griscelli-Prunieras: a propósito de dos casos. An Pediatría.

[7] Gottesberge Amm zum (1988). Physiology and Pathophysiology of Inner Ear Melanin. Pigment Cell Res.

[8] Murillo-Cuesta S, Contreras J, Zurita E, Cediel R, Cantero M, Varela-Nieto I (2010). Melanin precursors prevent premature age-related and noise-induced hearing loss in albino mice. Pigment Cell Melanoma Res.

[9] Guillery RW, Hickey TL, Kaas JH, Felleman DJ, Debruyn EJ, Sparks DL (1984). Abnormal central visual pathways in the brain of an albino green monkey (Cercopithecus aethiops). J Comp Neurol.

[10] Perez-Carpinell J, Capilla P, Illueca C, Morales J (1992). Vision defects in albinism. Optom Vis LWW.

[11] Grant S, Patel NN, Philp AR, Grey CN, Lucas RD, Foster RG (2001). Rod photopigment deficits in albinos are specific to mammals and arise during retinal development. Vis Neurosci.

[12] Garipis N, Hoffmann K-P (2003). Visual field defects in albino ferrets (Mustela putorius furo). Vision Res.

[13] Mártinez-García M, Montoliu L (2013). Albinism in Europe. J Dermatol..

[14] Lerner A, Fitzpatrick TB (1950). Biochemistry of melanin formation. Physiol Rev.

[15] Oetting WS, King RA (1994). Molecular basis of oculocutaneous albinism. J Invest Dermatol.

[16] Aigner B, Besenfelder U, Müller M, Brem G (2000). Tyrosinase gene variants in different rabbit strains. Mamm Genome.

[17] Schmutz SM, Berryere TG, Ciobanu DC, Mileham AJ, Schmidtz BH, Fredholm M (2004). A form of albinism in cattle is caused by a tyrosinase frameshift mutation. Mamm Genome.

[18] Blaszczyk WM, Arning L, Hoffmann KP, Epplen JT (2005). A Tyrosinase missense mutation causes albinism in the Wistar rat. Pigment Cell Res.

[19] Imes DL, Geary LA, Grahn RA, Lyons LA (2006). Albinism in the domestic cat (Felis catus) is associated with a tyrosinase (TYR) mutation.

[20] Blaszczyk WM, Distler C, Dekomien G, Arning L, Hoffmann KP, Epplen JT (2007). Identification of a tyrosinase (TYR) exon 4 deletion in albino ferrets (Mustela putorius furo). Anim Genet.

[21] Polanowski AM, Robinson-Laverick SM, Paton D, Jarman SN (2012). Variation in the tyrosinase gene associated with a white humpback whale (Megaptera novaeangliae). J Hered.

[22] Damé MCF, Xavier GM, Oliveira-Filho JP, Borges AS, Oliveira HN, Riet-Correa F (2012). A nonsense mutation in the tyrosinase gene causes albinism in water buffalo. BMC Genet.

[23] Ding B, Ryder OA, Wang XX, Bai SC, Zhou SQ, Zhang YP (2000). Molecular basis of albinism in the rhesus monkey. Mutat Res - Fundam Mol Mech Mutagen.

[24] Prado-Martinez J, Hernando-Herraez I, Lorente-Galdos B, Dabad M, Ramirez O, Baeza-Delgado C (2013). The genome sequencing of an albino Western lowland gorilla reveals inbreeding in the wild. BMC Genomics.

[25] Lynch Alfaro JW, Boubli JP, Olson LE, Di Fiore A, Wilson B, Gutiérrez-Espeleta GA (2012). Explosive Pleistocene range expansion leads to widespread Amazonian sympatry between robust and gracile capuchin monkeys. J Biogeogr.

[26] Lynch Alfaro JW, Izar P, Ferreira RG (2014). Capuchin monkey research priorities and urgent issues. Am J Primatol.

[27] Preising MN, Forster H, Gonser M, Lorenz B (2011). Screening of TYR, OCA2, GPR143, and MC1R in patients with congenital nystagmus, macular hypoplasia, and fundus hypopigmentation indicating albinism. Mol Vis.

[28] Untergasser A, Cutcutache I, Koressaar T, Ye J, Faircloth BC, Remm M (2012). Primer3-new capabilities and interfaces. Nucleic Acids Res.

[29] Hall T. BioEdit: a user-friendly biological sequence alignment editor and analysis program for Windows 95/98/NT Nucleic Acids Symp. Ser. London. 1999;41:95–8.

[30] Groves CP. Primate Taxonomy. Washington: Smithsonian Institution Press; 2001.

[31] Silva Jr J de S. Especiação nos macacos-prego e caiararas, gênero Cebus Erxleben, 1777 (Primates, Cebidae). Rio Janeiro Univ. Fed. do Rio Janeiro. 2001.

[32] Rylands AB, Kierulff MC, Mittermeier RA, Rylands AB, Kierulff MC, Mittermeier RA. Notes on the taxonomy and distributions of the tufted capuchin monkeys (Cebus, Cebidae) of South America. Lundiana. 2005;6:97–110.

[33] Lima MG, Buckner JC, Silva Jr J de S, Aleixo A, Martins A, Boubli JP, et al. Capuchin monkey biogeography: understanding Sapajus Pleistocene range expansion and the current sympatry between *Cebus *and *Sapajus*. J Biogeogr. 2017;44:810–20.

[34] Lanfear R, Calcott B, Ho SYW, Guindon S. PartitionFinder: Combined selection of partitioning schemes and substitution models for phylogenetic analyses. Mol Biol Evol. 2012;29:1695–701. Oxford University Press. 10.1093/molbev/mss020 22319168

[35] Ronquist F, Teslenko M, Van Der Mark P, Ayres DL, Darling A, Höhna S, et al. Mrbayes 3.2: Efficient bayesian phylogenetic inference and model choice across a large model space. Syst Biol . 2012;61:539–42. Oxford University Press. 10.1093/sysbio/sys029 PMC3329765 22357727

[36] Boubli JP, Rylands AB, Farias IP, Alfaro ME, Alfaro JL. Cebus Phylogenetic Relationships: A Preliminary Reassessment of the Diversity of the Untufted Capuchin Monkeys. Am J Primatol. 2012;74:381–93. 10.1002/ajp.21998 22311697

[37] Alfaro JWL, Silva J de SE, Rylands AB. How Different Are Robust and Gracile Capuchin Monkeys? An Argument for the Use of Sapajus and Cebus. Am J Primatol. 2012;74:273–86. 10.1002/ajp.22007 22328205

[38] Giebel LB, Musarella MA, Spritz RA. A nonsense mutation in the tyrosinase gene of Afghan patients with tyrosinase negative (type IA) oculocutaneous albinism. J Med Genet. 1991;28:464–7. 10.1136/jmg.28.7.464 PMC1016956 1832718

[39] Anistoroaei R, Fredholm M, Christensen K, Leeb T (2008). Albinism in the American mink (Neovison vison) is associated with a tyrosinase nonsense mutation. Anim Genet..

[40] Schneider H, Bernardi JAR, da Cunha DB, Tagliaro CH, Vallinoto M, Ferrari SF (2012). A molecular analysis of the evolutionary relationships in the Callitrichinae, with emphasis on the position of the dwarf marmoset. Zool Scr..

[41] Irwin DM, Kocher TD, Wilson AC (1991). Evolution of the cytochrome b gene of mammals. J Mol Evol..

[42] Rylands AB, Mittermeier RA, Bezerra BM, Paim FP, Queiroz HL. Species accounts of Cebidae. In: Mittermeier RA, Rylands AB, Wilson DE, editors. Handbook of the Mammals of the. World - Vol. 3 Primates. Barcelona: Lynx Edicions; 2013. p. 952.

