# Supplementary material for: A novel nonsense mutation in the tyrosinase gene is related to the albinism in a capuchin monkey (Sapajus apella)
Source: BMC Genet. 2017 May 5;18:39. doi: 10.1186/s12863-017-0504-8 (PMC5420114; doi:10.1186/s12863-017-0504-8)
Supplement: Supplementary file 1 — GenBank accession numbers and geographic coordinates for robust capuchin samples from GenBank used in this study. (PDF 95 kb) [file 12863_2017_504_MOESM1_ESM.pdf]

SUPPLEMENTARY TABLE 1: GenBank accession numbers and geographic coordinates for robust capuchin samples from GenBank used in this study.

**Sample N° (from Lima et al., 2016, in press)**	**Species (Rylands et *al.,* 2013)**	**Subclade Assignment for *Sapajus* CLADE 4 (Lima et al., 2016, in press)**	**LAT**	**LONG**	**GenBank Accession N° Cyt *b***	**GenBank Accession N° Dloop**
1	*S. flavius*	1	-6.56	-35.13	KY173296	KY173187
2	*S. flavius*	1	-7.01	-34.96	KY173297	KY173188
3	*S. flavius*	1	-7.02	-35.09	KY173298	KY173189
4A	*S. xanthosternos*		-14.79	-39.05	KY173340	KY173233
4B	*S. xanthosternos*		-14.79	-39.05	KY173341	KY173234
5	*S. xanthosternos*		-15.17	-39.07	KY173343	KY173236
6	*S. xanthosternos*		-15.41	-39.50	KY173342	KY173235
C	*S. robustus*		-	-	KY173339	KY173231
15	*S. nigritus*		-23.94	-54.22	KY173338	KY173230
17	*S. libidinosus*	2	-2.77	-41.81	KY173300	KY173191
18	*S. libidinosus*	2	-2.8	-41.87	KY173301	KY173192
19	*S. libidinosus*	2	-2.85	-41.83	KY173302	KY173193
20	*S. libidinosus*	2	-5.09	-42.43	KY173299	KY173190
21	*S. libidinosus*	2	-7.93	-44.20	KY173303	KY173194
22	*S. libidinosus*	2	-5.28	-48.30	KY173304	KY173195
23A	*S. libidinosus*	2	-14.14	-48.17	KY173307	KY173197
23B	*S. libidinosus*	2	-14.14	-48.17	KY173306	KY173198
26	*S. libidinosus*	2	-16.6	-49.26	KY173308	KY173199
27	*S. libidinosus*	2	-16.11	-50.30	KY173305	KY173196
29	*S. cay*	3	-16.06	-57.72	KY173295	KY173186
32A	*S. apella*	2	-6.15	-49.56	KY173275	KY173166
32B	*S. apella*	2	-6.15	-49.56	KY173276	KY173167
33A	*S. apella*	2	-3.83	-49.64	KY173259	KY173151
33B	*S. apella*	2	-3.83	-49.64	KY173255	KY173147
33C	*S. apella*	2	-3.83	-49.64	KY173256	KY173148
33D	*S. apella*	2	-3.83	-49.64	KY173258	KY173150
33E	*S. apella*	2	-3.83	-49.64	KY173257	KY173149
41	*S. apella*	5	3.22	-52.03	KY173253	KY173145
42	*S. apella*	5	0.83	-53.93	KY173266	KY173158
43	*S. apella*	5	0.63	-55.73	KY173268	KY173160

44A	*S. apella*	5	-0.17	-55.19	KY173263	KY173155
44B	*S. apella*	5	-0.17	-55.19	KY173264	KY173156
45	*S. apella*	5	-0.96	-55.52	KY173262	KY173154
47	*S. apella*	5	1.29	-58.70	KY173265	KY173157
49	*S. apella*	5	-1.92	-59.47	KY173290	KY173181
52	*S. apella*	5	-2.47	-58.40	KY173288	KY173179
53	*S. apella*	5	-2.6	-56.18	KY173277	KY173168
54	*S. apella*	5	-3.18	-55.80	KY173282	KY173173
59A	*S. apella*	4	-9.6	-56.01	KY173285	KY173176
59B	*S. apella*	4	-9.6	-56.01	KY173286	KY173177
59C	*S. apella*	4	-10	-56.04	KY173283	KY173174
59D	*S. apella*	4	-10	-56.04	KY173284	KY173175
61	*S. apella*	3	-12.56	-63.44	KY173281	KY173172
62	*S. apella*	3	-12.5	-63.53	KY173293	KY173184
63	*S. macrocephalus*	5	-3.37	-60.48	KY173313	KY173204
64	*S. macrocephalus*	5	-4.44	-60.32	KY173312	KY173203
66	*S. macrocephalus*	6	-4.86	-61.41	KY173324	KY173216
67A	*S. macrocephalus*	6	-4.99	-62.96	KY173329	KY173221
67B	*S. macrocephalus*	6	-4.99	-62.96	KY173327	KY173219
68	*S. macrocephalus*	6	-5.69	-63.24	KY173328	KY173220
71A	*S. macrocephalus*	6	-8.89	-63.24	KY173260	KY173152
71B	*S. macrocephalus*	6	-8.89	-63.24	KY173261	KY173153
71C	*S. macrocephalus*	6	-8.89	-63.24	KY173310	KY173201
72	*S. macrocephalus*	6	-8.67	-62.37	KY173332	KY173224
75	*S. macrocephalus*	5	-2.47	-64.83	KY173318	KY173209
76	*S. macrocephalus*	6	-2.59	-64.89	KY173319	KY173210
77A	*S. macrocephalus*	6	-2.45	-65.36	KY173320	KY173211
78	*S. macrocephalus*	5	-1.05	-62.89	KY173316	KY173207
79	*S. macrocephalus*	5	-0.48	-64.41	KY173315	KY173206
80	*S. macrocephalus*	5	-0.61	-64.92	KY173330	KY173222
81	*S. macrocephalus*	5	-0.23	-66.85	KY173325	KY173217
82	*S. macrocephalus*	5	-1.84	-69.03	KY173326	KY173218
85	*S. macrocephalus*	6	-4.4	-70.14	KY173317	KY173208
