# Supplementary figures and images for: A novel nonsense mutation in the tyrosinase gene is related to the albinism in a capuchin monkey (Sapajus apella)
Source: BMC Genet. 2017 May 5;18:39. doi: 10.1186/s12863-017-0504-8 (PMC5420114; doi:10.1186/s12863-017-0504-8)

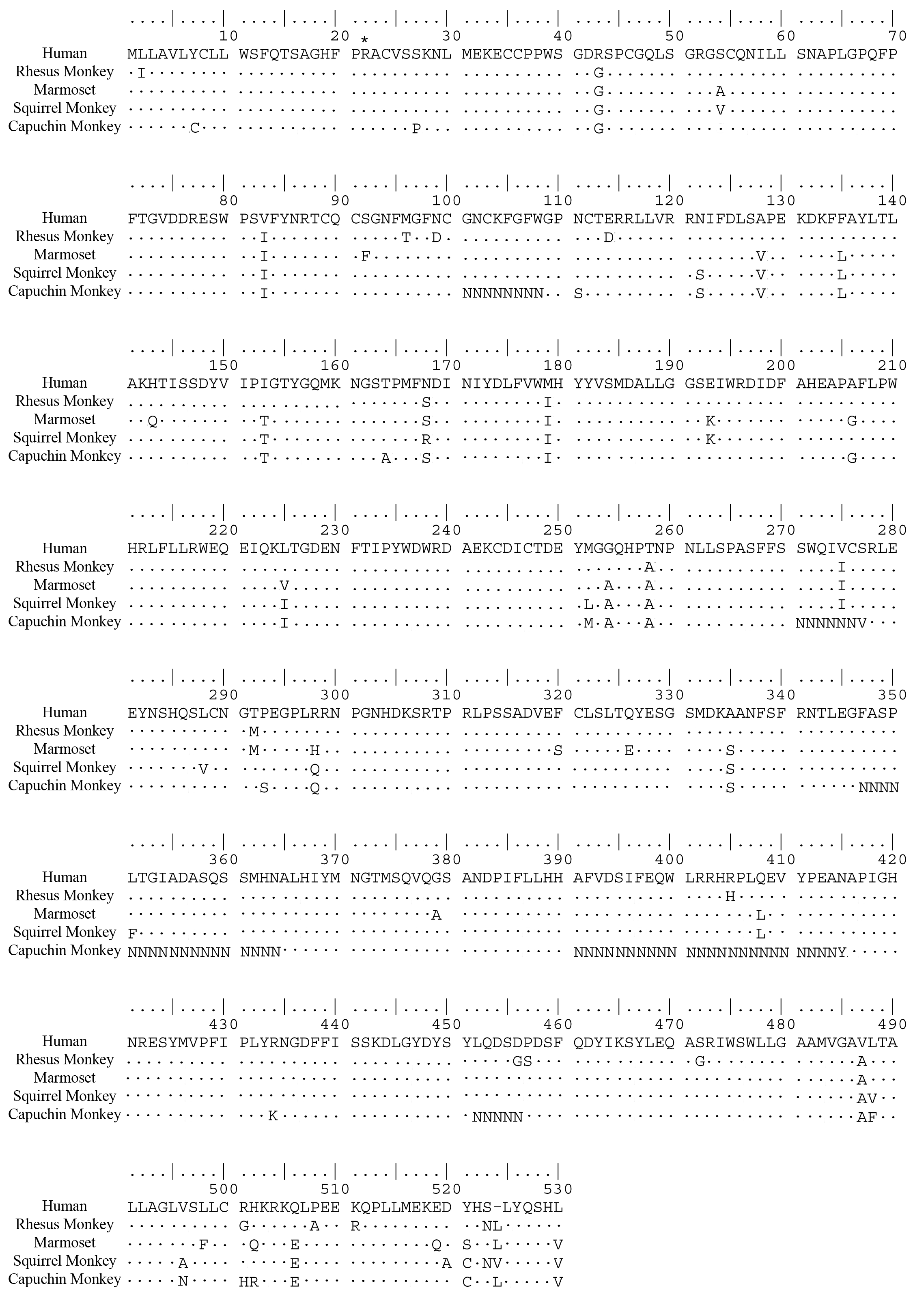

Supplement: Supplementary file 2 — Alignment of the amino acid sequences of the TYR proteins of human (XM_011542970.2), rhesus monkey (XM001105033.3), marmoset (XM_002754705.2), squirrel monkey (XM_003935082.1) and the capuchin monkey. Identical residues are indicated by dots. The position of the nonsense mutation in the albino capuchin monkey, Sivuca, is shown by a star. (TIF 1166 kb) [file 12863_2017_504_MOESM2_ESM.tif]
